# Alcohol use and breast cancer risk: A qualitative study of women’s perspectives to inform the development of a preventative intervention in breast clinics

**DOI:** 10.1111/ecc.13075

**Published:** 2019-04-30

**Authors:** Sophia E. Chambers, Ellen R. Copson, Peter F. Dutey‐Magni, Caspian Priest, Annie S. Anderson, Julia M.A. Sinclair

**Affiliations:** ^1^ Clinical and Experimental Sciences, Faculty of Medicine University of Southampton Southampton UK; ^2^ Cancer Sciences, Faculty of Medicine University of Southampton and University Hospital Southampton Southampton UK; ^3^ Institute of Health Informatics University College London London UK; ^4^ Centre for Research into Cancer Prevention and Screening, Ninewells Hospital & Medical School University of Dundee Dundee UK

**Keywords:** alcohol, breast cancer, intervention, patient information, prevention, qualitative

## Abstract

**Objective:**

This study aimed to explore women's views about breast cancer risk and alcohol use, to inform the design of a prototype for an intervention in breast clinics about alcohol as a modifiable risk factor for breast cancer.

**Methods:**

Women recruited in NHS breast screening and symptomatic clinics in Southampton, UK, were invited to take part in semi‐structured telephone interviews or a focus group to discuss their perspectives of breast cancer risk, alcohol consumption and their information needs about these topics. Data were analysed thematically. Twenty‐eight women took part in telephone interviews, and 16 attended one of three focus groups.

**Results:**

While most women reported a personal responsibility for their health and were interested in advice about modifiable risk factors, few without (or prior to) experience of breast symptoms independently sought information. Many considered alcohol advice irrelevant as the association with breast cancer was largely unknown, and participants did not consider their drinking to be problematic. Women reported trusting information from health organisations like the NHS, but advice needs to be sensitive and non‐blaming.

**Conclusion:**

NHS breast screening and symptomatic clinics offer a “teachable moment” to engage women with context‐specific advice about alcohol and cancer risk that, if targeted correctly, may assist them in making informed lifestyle choices.

## 1 BACKGROUND

In the UK, around 54,800 women are diagnosed with breast cancer annually (Cancer Research UK, 2018). The latest World Cancer Research Fund and American Institute for Cancer Research (2018) Continuous Update Project report found “strong evidence” that alcohol consumption is a probable cause of premenopausal breast cancer, and a convincing cause of post‐menopausal breast cancer, with a 5%–9% increased risk per 10 g ethanol per day. However, knowledge of alcohol as a risk factor for breast cancer is low, with <20% of the general public (Buykx et al., 2016) and women attending breast clinics (Sinclair et al., 2018) in England, able to identify the association unprompted.

There has been some exploration of the provision of alcohol advice to patients following a diagnosis of cancer (Simapivapan, Hodge, & Boltong, 2018; Williams, Beeken, Fisher, & Wardle, 2015). However, as prevention is the most cost‐effective long‐term strategy for cancer control, there is a need to educate and empower individuals prior to diagnosis, to make informed lifestyle choices to moderate their cancer risk (World Health Organisation, 2014). Breast screening and symptomatic clinics can present ideal “teachable moments” to give prevention advice (Evans & Howell, 2015), with existing lifestyle interventions in this setting demonstrating good uptake and receiving positive feedback (Anderson et al., 2014; Macleod & Anderson, 2018). Recent work from our group showed that women were receptive to adding a brief “cancer prevention information session” to screening or symptomatic clinics (Sinclair et al., 2018). However, research also highlights factors that may make communication about modifiable risk factors challenging, including beliefs about the role of luck and genetics in developing cancer (Conway, Wyke, Sugden, Mutrie, & Anderson, 2016; Dumalaon‐Canaria, Hutchinson, Prichard, & Wilson, 2014; Wright et al., 2015).

To enhance the acceptability and feasibility of an intervention, it needs to be tailored to the context and needs of the target population (Yardley, Morrison, Bradbury, & Muller, 2015). Consideration of patient perspectives during the feasibility phase of intervention development can maximise effectiveness and protect against the development of inappropriate or unacceptable interventions (Craig et al., 2008; Wight, Wimbush, Jepson, & Doi, 2015). The UK Medical Research Council provided funding to undertake the formative work needed to design and develop a prototype for a brief alcohol intervention, for women attending breast clinics. Using qualitative methods, we were interested in exploring women's views in relation to breast cancer risk, alcohol consumption and their information needs about these topics.

## 2 METHODS

### 2.1 Participants and setting

Participants were recruited as part of the “Abreast of Health Intervention Development Study,” designed to ascertain the acceptability and feasibility of delivering an alcohol brief intervention in breast clinic settings. Women aged 18+ who had attended a breast appointment (symptomatic or screening) in the last 2 years were invited to attend a focus group or take part in a telephone interview. Recruitment was by posters/postcards in clinic areas inviting eligible participants to contact the research team. Participants from another phase of the study (a web‐based survey of clinic attenders) were also invited to participate. Participants were told they would be asked to give their opinion about advice on modifying the risk of developing breast cancer and possible methods for delivering this information. All participants gave informed consent to take part and for interviews/focus groups to be audio recorded. The appropriate NHS Ethics Committee approved the study (Reference: 17/LO/0953).

Three focus groups (total *n* = 16) and 28 telephone interviews were conducted with women ≥18 years who had attended a symptomatic breast clinic (72.7%) or an NHS breast screening programme mammogram (27.3%) within the past 2 years.

### 2.2 Data collection and analysis

Interviews and focus groups were based on one of three topic guides which covered themes relevant to intervention development (see Table S1); one focus group was conducted per topic guide, with the number of participants indicated in Table 1. The sample size (maximum of 10 interviewees and one focus group per topic guide) was chosen on the grounds that this was practical and realistic to maximise the range of views within the “Abreast of Health” study timescale.

**Table 1:** Topic guide description with corresponding number of telephone interviews and focus group participants

Topic guide description	Telephone interviews (*n*)	Focus group participants (*n*)
1. “Breast cancer risks”: to gather participants’ knowledge and views about causes and risks of breast cancer, and gauge levels of awareness of modifiable risk factors, including alcohol.	9	3
2. “Alcohol consumption”: to understand how women talk about their relationship with alcohol and view the role of alcohol in society and in relation to health.	9	3
3. “Information needs”: to explore the relative importance women give to knowing about modifiable risk factors for breast cancer. To understand when and why women would seek information relating to breast health, and identify the types of information they seek, and from which sources.	10	10
Total number of participants	28	16

The focus group based on “information needs” also asked participants to test several existing cancer and alcohol information apps/websites (e.g. Public Health England's Drink Tracker, “Reduce My Risk” website) to facilitate discussion. Interviews lasted ~25 min and focus groups ~90 min.

Data were transcribed verbatim and transferred into the qualitative software programme, NVivo11, to facilitate analysis. Thematic analysis was used to explore and interpret patterns in the data, pertaining to the concepts listed above (Braun & Clarke, 2006). While the separate topic guides provided a structure for interview and focus group content, analysis involved moving back and forth between the entire dataset, as many women discussed issues relevant to several themes. The coding scheme was developed by SC, but reviewed by the wider research team to facilitate thorough and comprehensive analysis, and ensure other avenues of analysis were explored.

## 3 RESULTS

Most women said they have a personal responsibility for taking care of their health, and acquisition of reliable and accurate information is imperative in this process:

We have a responsibility to look after our bodies and so we need to be as well informed as we possibly can about what risk factors are, and how to keep ourselves healthy. (T125)

However, various tensions (both explicit and implicit) were identified in participants’ accounts which may challenge the effectiveness of an intervention seeking to educate women about alcohol as a major modifiable risk factor for breast cancer. Table 2 displays the challenges as relating to (a) perceptions of cancer risk, (b) views on alcohol consumption and (c) women's information needs. The following narrative expands upon each of these in turn.

**Table 2:** Challenges identified through analysis of women's accounts for the successful implementation of an alcohol‐focused intervention in breast clinics

Perceptions of cancer risk	Views on alcohol consumption	Information needs
A focus on symptomatology	Alcohol consumption as irrelevant to breast health	Knowing who and what to trust
Salience of personal experience	“That's not me”: othering in alcohol consumption	“Finger pointing”

### 3.1 Perceptions of cancer risk

#### 3.1.1 A focus on symptomatology

Analysis revealed a reactive, rather than preventative, approach to dealing with breast cancer: “People don't often discuss cancerous things…unless they've found something like a lump, it's just not spoken about” (TI22). This was echoed in participants’ passivity in seeking information to understand risk factors:

To be honest with you, I probably wouldn’t look anything up unless I had symptoms. (TI21)

When pressed for the kinds of information they would seek if they *were* interested in understanding more about breast health, most women agreed they would search (typically online) for “symptoms of breast cancer”; only a minority said they would search for “risks” or “causes.” However, when asked explicitly during interview the importance of knowing about risk factors, every participant stated it was “very” or “extremely” important, particularly “if there are definite things that you can change” (TI17). Nevertheless, despite this assertion, women's apparent bias towards a reactive approach to breast cancer may reduce their receptivity to information about making healthier lifestyle choices, particularly if they are asymptomatic: “I would read it for interest and discard it” (FG1, Pt02).

Without breast symptoms, women explained it can be easy to ignore health information: “If I feel healthy, I would continue on with what I'm doing rather than check” (TI27); “I'd just want to brush it under the carpet” (FG1, Pt01). One participant suggested this is particularly true of younger women:

When you need to know about it, you probably don’t want to know about it, which is when you’re a lot younger…you don’t think about the consequences because you’re so young at the time. (TI21)

Avoidance of information was also associated with fear of cancer (“if I was really scared, I probably wouldn't look,” TI19; “you can scare yourself into thinking that there's a problem”, TI23), and other times, invincibility (“most people don't believe they will ever get cancer,” TI26). Attendance at a breast clinic was therefore described as a window of opportunity for learning about risk factors, as concerns for breast health are made salient:

I went to this appointment; now I believe it’s important [to know about risk factors], but before, it was something I didn’t really think about because it was just not on my radar. (TI16)

#### 3.1.2 Salience of personal experience

Personal and/or familial experience appeared to have a particularly influential role in the formation of beliefs about the causes of, and risk factors for, breast cancer. While participants identified numerous factors that may lead to cancer generally, those with lived experience of breast cancer often attributed their diagnosis (or that of a close friend/relative) to one cause. For example:

I was on the [contraceptive] pill for X number of years and I strongly believe that my oestrogen pill fed my cancer. (TI27)

The one thing that I do think has linked to the breast cancer, certainly in my sister’s case, is stress; massive, massive stress. (TI04)

Moreover, numerous participants detailed stories of individuals they knew who appeared to defy evidence about the risks of modifiable factors for developing cancer:

I know of people who are, eat totally bad, had a bad lifestyle foodwise, smoking, drinking, and they live until they’re 100. I also know people who are very healthy, never smoked in their life, never drank in their life, vegetarians, or whatever, and they still get cancer. (TI27).

The frequency with which participants described such cases suggests they carry significant weight in how risks for breast cancer are understood and evaluated. Importantly, anecdotal evidence appeared to reduce acceptance of “evidence‐based” information which did not match participants’ personal narratives, particularly that relating to modifiable factors: “What you said about the drinking, um, I've sort of, rarely drank…I still got cancer.” (FG3, Pt07). Similarly, women with familial history of breast cancer often described a cancer diagnosis as predetermined; “there's nothing you can do about it” (TI12), “it's what you're born with” (TI09). A fatalistic approach to breast cancer risk may therefore present as a challenge for women considering preventative information about factors such as alcohol consumption.

### 3.2 Views on alcohol consumption

#### 3.2.1 Alcohol consumption as irrelevant to breast health

There was an apparent bias against discussions around risky alcohol consumption within the context of breast health in several interviews and focus groups, which may challenge effective intervention delivery within clinical settings. While numerous participants recognised that “drinking too much alcohol is bad for your health” (T103), and concern about health was considered a valid reason for reduced consumption or abstinence, very few discussed alcohol in association with breast cancer risk specifically. In fact, several women appeared confused by questions relating to alcohol use within the context of breast cancer research:

Bearing in mind this is breast cancer research, I’d like to ask you why you’re concentrating on alcohol? (TI06)

I’m confused as to why this is a breast cancer care topic. (TI01)

Participants often dismissed the relevance of alcohol information for them personally (“I wouldn't even look at it [alcohol information] because I don't really drink,” FG3, Pt03), or as a topic for discussion within breast clinics:

If you’re sitting in a waiting clinic, your head isn’t exactly on…‘how much alcohol did I consume last week?’ It’s a, it’s a slightly alien distraction. (FG3, Pt08)

Another common theme was the belief that information about alcohol harms would be better directed at younger generations because of the perceived disparity in level of risk (“the biggest alcohol abuse is happening in young people,” TI12). Moreover, some women said alcohol interventions would be delivered “too late” for those attending symptomatic clinics as “the damage has been done” (FG3, Pt04). Thus, perceptions of alcohol information as being irrelevant for women at their stage of life may reduce their willingness to engage with such information in breast clinics. However, despite prior ignorance to the association between alcohol and breast cancer risk, many participants appeared interested to learn more as part of the research process:

I didn’t know until I saw you at the clinic that it was specifically one of the risk factors for breast cancer…I haven’t been looking, but I haven’t noticed any leaflets relating to alcohol and breast cancer anywhere. (TI21)

Several also explained how their participation had prompted discussions about alcohol with others in their life, which suggests a readiness to discuss the topic once initial barriers to doing so had been overcome:

I told my mum today. She’s a nurse. I was telling her about the research – she didn’t even know that [alcohol is a risk factor for breast cancer]. (TI05).

#### 3.2.2 “That's not me”: Othering in alcohol consumption

Analysis highlighted that entrenched discourses around what constitutes “normal drinking” in England may also impede discussions about alcohol consumption as a risk factor for breast cancer. Participants typically described problematic drinking as synonymous with dependent or “alcoholic” drinking and characterised by loss of control and severe harm. This appeared to generate an “othering” effect in which some participants considered themselves fundamentally different to those with an alcohol “problem” (FG2):

“Pt01: That’s an alcoholic, and that person will end up in the gutter and lose their home, and you know that’s kind of the extreme picture.

Pt02: That’s not me.

Pt01: Yeah exactly, that’s not me because I’m normal.”

Women's self‐defined identity as a “normal drinker,” characterised by sociability and personal control, even if that is at levels that increase the risk of breast cancer, may hinder efforts to engage them in discussions about making changes to their consumption. Indeed, in discussing the alcohol‐specific digital apps explored during a focus group, one participant said:

If you had an alcohol problem, then yes, they [the apps] were very useful…I would suggest that it wasn’t necessarily relevant to where we’re coming from. (FG3, Pt04)

Nevertheless, several participants reported “lying” to medical professionals about the number of alcohol units they consumed, or more commonly, admitted they were either unsure of current national drinking guidelines, or paid little attention to them. This highlights a gap in participants’ knowledge about alcohol consumption beyond its associated risk for breast cancer (the UK lower risk drinking level is 14 units or 112 g of ethanol per week). Information given as part of an alcohol intervention in breast health setting therefore needs to make salient its relevance for individuals who are drinking at levels that increase their risk for breast cancer, but are not aware of this, and do not consider their drinking problematic. Indeed, several participants reported a willingness to share alcohol‐related information to others for whom they perceived needed it more: “I would happily send on to all the young people I know” (FG3, Pt08).

### 3.3 Women's information needs

#### 3.3.1 Knowing who and what to trust

Participants described a landscape of mixed messages about risk factors for cancer, and the subsequent difficulty in obtaining accurate and reliable health information: “the advice you're given constantly changes, so it's sort of, quite fickle” (TI17); “you get so much conflicting information [about modifiable risk factors for cancer] that you don't know which way to turn” (TI12). Women most often referred to the NHS, healthcare professionals, or breast cancer charities as trustworthy sources of information. Those who reported using the Internet tended to limit their search to websites provided by these sources: “I only look at the NHS websites because I don't trust most of the internet” (FG1, Pt03).

The information imparted by researchers and scientists was often considered less reliable, and rarely taken at face value:

Often you get things reported, and you think, ‘oh wow, that’s fantastic’, and then you find out that they’ve just done research on mice, or it’s been in a petri dish. (TI13)

I’d want to see the full research…show me your study and how it’s been managed and how you’ve come to these conclusions. (TI24)

Generally, participants were only convinced by research that had been vetted by a “respected” organisation such as the NHS, or had been conducted by a clinician with expertise in that area of health: “if there were doctors that I was aware of that had actually written the article or contributed to the article, or the study, or the research, then that would be a big plus” (TI19).

Although participants often read health‐related information communicated in the media (television, magazine, social media), they unanimously agreed it cannot be trusted or taken seriously:

I would take it with a pinch of salt because there’s so much information nowadays on the internet and in the media, social media and that, fake news. I’d want it backed up by information on the NHS websites. (TI23)

Despite this, many women reported discussing information they had seen in the media with female peers and family members: “We gossip…I would say something like, ‘did you see such‐and‐such or did you read this magazine article?’” (TI19). Indeed, some participants confirmed “hearsay” to be the main source of their knowledge about risk factors for breast cancer:

“Interviewer: How do you know what is a real risk factor?

Participant: Only from what you hear, and from other people.” (TI22)

With inconsistent media messages about modifiable risk factors such as alcohol consumption, women highlighted the difficulty in navigating information to inform their decisions about lifestyle choices:

Companies are very good at making these alcoholic beverages appealing to people, especially the ‘a glass of wine, red wine, a day is good for you’. They’re so clever, aren’t they? (T105)

#### 3.3.2 “Finger pointing”

A final theme running throughout the data was the need for information about risk factors that is not pejorative or blaming towards women for making “bad” lifestyle choices: “that's where the vilification comes in…we are just trying to lead a normal life, and maybe we'll have a glass of wine every now and again, and we are being told that's wrong” (FG3, Pt04). Several women spoke of the emotional sequelae of attending a breast clinic or experiencing breast symptoms, and the need for this to be considered when communicating health information:

It’s a really emotional thing…so I guess somehow in the communications or information, making sure we’re really aware of the emotions that people may be going through and make sure we’re sensitive and responsive to that. (TI26)

There was a preference for messages that incorporated clear facts about risks but with an acknowledgement that scientific knowledge is incomplete, and numerous, intertwining factors can contribute to one's risk of cancer. Participants alluded to the importance of language in communicating such information, with several averse to definitive statements about “causes” of cancer which could “create a blame culture”:

I don’t believe that you’re in the situation at the moment that anyone can categorically put their finger on something and say this is exactly what caused it; I think the term ‘contributing factor’ is far more reasonable. (TI12)

The same participant suggested that subtle changes in terminology can have a profound impact on women's receptivity to breast health information, a point echoed in several other interviews: “by changing the terminology…you're removing yourself from the finger pointing aspect of it.”

Participants also discussed the need to respect an individuals’ decision for not taking steps to change their lifestyle when it may reduce their risk of cancer: “I know I drink too much, and I know that potentially could be a risk factor but that's a considered choice on my behalf” (TI24). Several described a “balancing act” between taking responsibility for optimising one's health longer term, and their prerogative to make lifestyle choices on a given day. However, in order to manage this tension, women agreed that they need impartial and personally relevant information:

The balance comes if the risks are strong enough, and if I’m aware enough…I would evaluate that on conversations with health professionals in terms of my own personal risk factors. (TI25)

## 4 DISCUSSION

Health events, such as cancer screening or investigation of symptoms, are considered ideal “teachable moments” for prevention interventions, as they can optimise already‐heightened patient motivation into changing risk‐inducing behaviours like alcohol consumption (Anderson, Mackison, Boath, & Steele, 2013; Evans & Howell, 2015; Lawson & Flocke, 2009; Senore, Giordano, Bellisario, Di Stefano, & Segnan, 2012). To inform the development of a “person‐based” (Yardley et al., 2015) intervention to embed prevention advice within breast clinics, this study explored women's perspectives of breast cancer risk and alcohol consumption. Analysis of interview and focus group data highlighted challenges (and some potential solutions) to women engaging with alcohol brief advice given in this context, which needs to be considered to optimise the acceptability and effectiveness of any intervention.

Although participants affirmed the importance of obtaining reliable information to make informed choices about health, there was often a disjuncture with their self‐reported efforts to do so. Several women, especially those without (or prior to) experience breast cancer symptoms, reported no attempts to seek information about risk factors, or disregarded information they had found; this was often due to perceptions that such information was irrelevant, fear‐inducing or pejorative. Moreover, participants who expressed fatalistic views about cancer appeared more resistant to information regarding modifiable risk factors—and recent research found an association of such views with engagement in unhealthy behaviours (Anderson et al., 2017). An awareness of how different perspectives of cancer (such as those identified in the present study) may affect women's receptivity to preventative interventions, can help researchers and clinicians reduce barriers to engagement (Conway et al., 2016).

Around 20% of women aged 45–64 in England (16% across all ages) drink above the UK Chief Medical Officer's current guidelines of 14 units of alcohol per week (Health & Social Care Information Centre, 2017). However, participants in the present study did not consider their drinking as problematic and typically discussed risky drinking within the context of “alcoholism” or excessive/binge drinking within younger people (although only 15% of 16–24‐year‐old drink more than 14 units per week) (Health & Social Care Information Centre, 2017); many therefore questioned the relevance of alcohol advice in relation to health risks of their “unproblematic” levels of consumption. Accepting advice about drinking levels may also be made more challenging by the social and cultural normativity of alcohol consumption (Bartram, Eliott, & Crabb, 2017; Piacentini & Banister, 2009). While research has found that many patients are interested in receiving lifestyle advice at the time of breast cancer screening (Fisher, Dowding, Pickett, & Fylan, 2007; Fisher, Wilkinson, & Valencia, 2016), alcohol may be a difficult topic to address. Indeed, a recent study with adults eligible for breast, bowel or cervical cancer screening in England found lower patient willingness to receive advice about alcohol compared to physical activity, weight and diet (32% vs. 62%–67%) (Stevens, Vrinten, Smith, Waller, & Beeken, 2018).

Nevertheless, despite some initial hesitancy to engage in discussions about alcohol, most participants in the present study were interested to learn of its association with breast cancer and appeared willing to share this information with female friends and family members. Integrating alcohol advice within a broader healthy lifestyle intervention may be one way to increase women's receptivity, although this needs further investigation. Moreover, participants’ mistrust of much information about breast cancer and lifestyle, with the exception of that provided by the NHS, suggests that NHS breast clinics provide an ideal setting in which to deliver such advice, and facilitate discussions that women might ordinarily be reluctant to have. Supporting this, Stead, Caswell, Craigie, Eadie, and Anderson (2012) argue that where the association between a modifiable risk factor and cancer is not naturally present in patients’ minds, healthcare professionals can play an important role in making these links explicit. However, given the emotional and anxiety‐provoking effects of attending breast clinics and screening (Brett, Bankhead, Henderson, Watson, & Austoker, 2005; Montgomery & McCrone, 2010; Woodward & Webb, 2001), any communication of health information needs to be non‐blaming and sensitive.

### 4.1 Strengths and limitations

The results presented in this paper reflect the views of a small number of women seen in screening and symptomatic clinics in one city in the UK. While this is broadly the target population for whom our intervention is being designed, these findings may not be generalisable to other parts of the UK. Moreover, all participants were willing and able to discuss the topics of breast cancer and alcohol, and although not systematically recorded, several participants reported personal experience of cancer, which may have had an impact on the opinions given. This is an important consideration as only ~10% of women attending symptomatic clinics, and <1% attending screening clinics in the UK, receive a breast cancer diagnosis (Adams, Midha, Postulka, & Ortiz, 2015; Health & Social Care Information Centre, 2018). Finally, although our data is too limited to perform subgroup analysis, participants recruited via symptomatic and screening clinics may represent two distinct groups whose views differ about the prospect of receiving a brief alcohol intervention, and this requires further investigation.

## 5 CONCLUSION

Women reported a personal responsibility for their health and expressed interest in learning about modifiable risk factors for breast cancer, especially from trusted organisations such as the NHS. Breast screening and symptomatic clinics present an opportunity for a “teachable moment” for preventative breast cancer advice, supporting prior research in this setting, for example (Anderson et al., 2014; Conway et al., 2016; Macleod & Anderson, 2018; McLeish et al., 2013). Nevertheless, the specific challenges in communicating information about alcohol as a modifiable risk factor will need to be considered when developing an alcohol‐focused intervention for women attending breast clinics.

## CONFLICT OF INTEREST

None for any author.
