# Supplementary material for: Alcohol use and breast cancer risk: A qualitative study of women’s perspectives to inform the development of a preventative intervention in breast clinics
Source: Eur J Cancer Care (Engl). 2019 Apr 30;28(4):e13075. doi: 10.1111/ecc.13075 (PMC6767031; doi:10.1111/ecc.13075)
Supplement: Supplementary file 1 [file ECC-28-na-s001.docx]

**SUPPLEMENT**

**S1: Topic guides used for interviews and focus groups**

| **Topic guide** | **Questions** (and prompts) |
| --- | --- |
| “Breast cancer risks” | 1. **What do you think of when you hear the phrase “risk factors for breast cancer”?**  - Are there different types of risk factor? - What are the differences (if any) between a risk factor and a cause? - Can you change/influence risk factors? - How do you know what is a real risk factor?  1. **How important is it to you personally to know about risk factors for breast cancer?**  - Does it depend on the type of factor (i.e. something you can change vs. something you can’t)? - Do you think people differ about what they want to know? - What impact would knowing about these things have – would you change your behaviour? - How do you balance managing risks with doing what you want?  1. **Do you have any other experiences or comments that might help us understand more about how people think and talk about risks factors for breast cancer?** |
| “Alcohol consumption” | 1. **Can you think of any reasons why people might drink?**  - Prompt examples: stress, boredom, taste… - Are women’s reasons different from men’s reasons? In what way?  1. **Can you think of any reasons why people might *not* drink alcohol?**  - Do you think these are valid reasons for not drinking? - If someone is not drinking alcohol – from your experience how do people who are drinking react? - Have you ever been to an alcohol-free party/wedding/event? What was it like?  1. **Can you give any examples of when people usually drink? Any particular occasions?**  - When are people expected to drink alcohol? Why do you think this is?  1. **How do you know when you have had a bit too much to drink?**  - Is it when you think, feel, or act in a certain way? - Does it depend on what type of alcohol you drink?  1. **What do we mean by “normal” drinking?**  - When does drinking become “abnormal”? - What does being “tee-total” / being “a drinker” mean? What does this impression give? - Is there any language we shouldn’t use when talking about alcohol consumption?  1. **Do you have any other experiences or comments that might help us understand more about how people think and talk about drinking alcohol in our society?** |
| “Information needs” | 1. **If you were worried or had a question about your breast health where would you go for information?**  - What would you type into ‘Google’ to get the information you need? - What would you hope to find out? - If you have ever sought out information, what was your experience of doing so? Did you get the answers you wanted? Was there anything that surprised you? - Was there any information you were given that you did not want? Why? - Are there reasons why you might not seek information?  1. **If you were to read an article - about say, breast cancer - how concerned are you about where the information has come?**  - Is it important for information to be accurate / reliable? Why? - How do you judge whether it is accurate / reliable? - What types of information do you trust? - What sources of information do you trust?  1. **If you came across some interesting / reliable info about risk factors for breast cancer, who in your life would you share that with? Why / why not?** (Mum, partner, daughter, friend…?) 2. **Do you have any other experiences or comments that might help us understand more about the information women want in relation to the risks associated with breast cancer?** |
